# Validity of Scores From the 12-Item Multiple Sclerosis Walking Scale Among Young, Middle-Aged, and Older Adults With Multiple Sclerosis

**DOI:** 10.1016/j.apmr.2025.09.023

**Published:** 2025-10-09

**Authors:** Robert W. Motl, Petra Šilić, Trinh L.T. Huynh, Brenda Jeng

**Affiliations:** a Department of Kinesiology and Nutrition, University of Illinois Chicago, Chicago, IL;; b Department of Medicine, University of Illinois Chicago, Chicago, IL.

**Keywords:** Aging, Cognition, Mobility, Patient-reported outcome measures, Rehabilitation, Validity, Walking

## Abstract

**Objective:**

To examine the construct validity of inferences from scores on the 12-item multiple sclerosis walking scale (MSWS-12) as a patient-reported outcome measure of walking dysfunction among young, middle-aged, and older adults with multiple sclerosis (MS).

**Design:**

Cross-sectional, comparative research design.

**Setting:**

University research laboratory.

**Participants:**

The sample included 187 participants (N=187) with MS who were categorized into young (20–39y, n=50), middle-aged (40–59y, n=83), and older (60–79y, n=54) adult age groups.

**Interventions:**

Not applicable.

**Main Outcome Measures:**

The participants completed the MSWS-12; the timed 25-foot walk, 6-minute walk, and timed Up and Go; and the Brief International Cognitive Assessment for MS. The data analyses were performed using IBM SPSS Version 29.0.2.0 for the iOS platform.

**Results:**

There were similar patterns of linear worsening of scores for the MSWS-12 (F_1,187_=12.7, *P*<.001, *ɳ*^2^=0.07), timed 25-foot walk (F_1,187_=11.5, *P*<.001, *ɳ* ^2^=0.06), 6-minute walk (F_1,187_=13.1, *P*<.001, *ɳ* ^2^=0.08), and timed Up and Go (F_1,184_=10.8, *P*<.001, *ɳ* ^2^=0.06) across the 3 age groups. MSWS-12 scores had strong partial correlations with timed 25-foot walk, 6-minute walk, and timed Up and Go in the overall sample (partial r’s of 0.59, 0.66, 0.55, respectively) and across the 3 age groups, when controlling for Brief International Cognitive Assessment for MS outcomes.

**Conclusions:**

We provide new evidence for the construct validity of MSWS-12 scores as a patient-reported outcome measure of walking dysfunction across the adult lifespan in MS. Our results, in particular, support the application of the MSWS-12 in clinical research and practice involving older adults with MS who experience the combined effects of advanced age and MS disease progression.

Multiple sclerosis (MS) is an immune-mediated, neurodegenerative disease of the central nervous system with an estimated prevalence of nearly 1 million adults in the United States.^1^ This disease typically has its onset in early or middle adulthood, but there is increasing evidence for MS as a disease with onset across the entire adult lifespan. MS is prevalent in young, middle-aged, and older adults, and the highest prevalence occurs among those 55 years of age and older.^1^ The high prevalence of MS among older adults is noteworthy, because it represents a condition of aging with a chronic disabling disease, and therefore reflects the overlap of advancing age and disease progression on manifestations of MS.^2^

One of the hallmark features of MS involves the loss of mobility,^3^ and this can be captured by performance-based walking outcomes such as the timed 25-foot walk (T25FW), 6-minute walk (6MW), and timed Up and Go (TUG).^4–6^ There is consistent evidence that performance on those tests is worse in MS than non-MS controls.^7,8^ There is further evidence that performance on those outcomes decreases across groups of young, middle-aged, and older adults with MS,^9^ and the difference in performance between MS and non-MS controls is consistent across the 3 age groups.^10^ Importantly, performance-based outcomes do not capture walking dysfunction from the perspective of the patient, and this has been a noteworthy limitation that motivated the development of patient-reported outcome measures (PROMs). PROMs are questionnaires that collect information on health outcomes directly from patients, including symptoms, health–related quality of life, and functional status.

Accordingly, there is value in measuring the loss of mobility based on the patient’s perspective,^11^ and this can be important as a screening instrument, descriptive measure, and an outcome within clinical trials that focus on improvement in walking from the patient’s perspective rather than performance outcomes alone. The 12-item Multiple Sclerosis Walking Scale (MSWS-12) was designed as a PROM of walking dysfunction over the past 2 weeks.^12^ The inference from MSWS-12 scores as reflecting walking dysfunction has been validated based on the expected strong pattern of correlations with scores from performance-based walking outcomes,^12^ and the correlations have been robust against cognitive dysfunction^13^ and elevated depressive and anxiety symptoms.^14^ We are unaware of research that has focused on the validity of inferences from MSWS-12 scores across the adult lifespan, particularly in samples of older adults with MS. Such inquiry is important for descriptive, cross-sectional, predictive, and experimental research involving adults with MS across the lifespan, because the perception of one’s physical capacity and actual walking ability can decline with increasing age and this might dissociate the expected pattern of age-related differences in scores on the MSWS-12 or its association with performance-based outcomes.

There are strong arguments for why scores from PROMs such as the MSWS-12 require validation across the lifespan, particularly among older adults with MS. There is evidence that domains of cognitive function decline with increasing age in MS,^15^ and cognitive dysfunction in older adults with MS might undermine the accurate recall of past experiences during everyday life tasks involving ambulation when rating items on the MSWS-12. There is further evidence of cognitive–motor coupling across the lifespan in MS, including older adults with MS,^16,17^ and this observation underscores the importance of controlling for cognition when examining the construct validity of the MSWS-12 scores. There is some evidence for changes in self-perceptions of physical capabilities with increasing age in MS,^18^ and this could present as a dissociation between self-perceptions of one’s ability and performance of physical tasks among older adults. If correct, such a dissociation could undermine the validity of MSWS-12 scores for capturing walking dysfunction in older adults with MS.

The validation of inferences from PROMs is an ongoing and evolving process,^19^ and the current study examined the construct validity of inferences from MSWS-12 scores as a PROM of walking dysfunction among young, middle-aged, and older adults with MS. This was undertaken using a historically derived framework involving a nomological network and hypothesis testing as fundamentals of establishing the construct validity of inferences from MSWS-12 scores.^19–23^ We first compared the 3 age groups for differences in MSWS-12 scores as well as performance-based walking outcomes as a known-group difference approach^19^ for construct validation of MSWS-12 scores. This analysis tested the hypothesis that older adults with MS would demonstrate worse MSWS-12 scores (ie, higher) than young and middle-aged adults with MS, and this would map with worse walking performance-based on the T25FW, 6MW, and TUG. We then examined bivariate correlations between MSWS-12 scores with performance-based walking outcomes and neurocognitive outcomes in the overall sample and 3 age groups as a convergent and divergent approach^19^ for construct validation of MSWS-12 scores. This analysis was based on a nomological network and tested the hypothesis that MSWS-12 would correlate more strongly with measures of walking performance (ie, T25FW, 6MW, and TUG) than measures of cognitive performance captured by the Brief International Cognitive Assessment for MS (BICAMS) in the overall sample and 3 age groups. The final analysis involved partial correlations between MSWS-12 scores with performance-based walking outcomes, controlling for neurocognitive outcomes to establish that neurocognitive status was not a source of bias in the construct validation framework. This analysis tested the hypothesis that MSWS-12 would correlate with measures of walking performance (ie, T25FW, 6MW, and TUG), even after accounting for cognitive performance (ie, BICAMS) in the overall sample and 3 age groups.

## Methods

### Participants and relevant procedures

The sample was recruited through word of mouth, flyers in the community, advertisements through the local National MS Society chapter, neurology clinics, and a therapeutic recreation facility for persons with disabilities. All participants underwent a phone screening for eligibility based on the following criteria: (1) MS diagnosis; (2) no relapse within the last 30 days; (3) age between 20 and 79 years; (4) ability to walk with or without assistive devices; and (5) willingness to complete the study protocol. We enrolled a sample of 187 participants with MS and categorized them into young (20–39y, n=50), middle-aged (40–59y, n=83), and older (60–79y, n=54) adult age groups.

The study protocol was approved by a University Institutional Review Board, and all participants provided written consent. Participants completed questionnaires regarding demographic and clinical characteristics as well as the MSWS-12, and underwent performance assessments of walking and neurocognitive function during a single visit in our laboratory.

### Measures

#### MSWS-12

The MSWS-12 contains 12 items rated on a 5-point scale with anchors of 1 (not at all) and 5 (extremely) based on the previous 2-week period.^12^ Overall MSWS-12 scores range between 0 and 100 points based on a scaled transformation (ie, sum item scores, subtract 12 from the sum, divide the remainder by 48, and multiply that value by 100) with higher scores reflecting greater perceived walking dysfunction.^12–14^

#### Walking performance

This study included 3 measures of walking performance because these outcomes should correlate strongly with MSWS-12 and support the convergent aspects of construct validity of inferences. The T25FW characterized walking performance-based on walking speed.^4^ The T25FW has strong psychometrics and real-world relevance in MS.^4^ The T25FW was administered based on standard instructions.^4^ The participant walked across a marked, 25-foot path as quickly and as safely as possible on 2 consecutive trials. The tester recorded the time per trial in seconds, and T25FW scores were averaged across the 2 trials.

The 6MW characterized walking performance-based on walking endurance.^5^ The 6MW has been well-characterized in MS^5^ and has strong psychometric properties in MS.^5^ The 6MW was administered using standard instructions for MS.^5^ The participant walked as far and as fast as possible during a 6-minute period, and the participant was allowed to take rests as needed.^5^ The tester recorded the total distance walked over the 6-minute period in feet.

The TUG characterized walking performance-based on functional walking mobility.^6^ On 2 trials, the participants stood up from a chair (without arm support), walked toward a cone placed 3 m ahead of the chair, turned around the cone, walked back to the chair, and then sat down (without arm support), as quickly and as safely as possible.^6^ The time per trial was recorded in seconds and then averaged across the 2 trials.

#### Neurocognitive performance

This study included the BICAMS battery^24^ because these outcomes should demonstrate small or moderate correlations with MSWS-12 and support the divergent aspects of construct validity of inferences. The BICAMS battery includes the Symbol Digit Modalities Test (SDMT), first 5 learning trials of the California Verbal Learning Test-II (CVLT-II), and first 3 learning trials of the Brief Visuospatial Memory Test-Revised (BVMT-R) for measuring information processing speed, verbal learning and memory, and visuospatial learning and memory, respectively.^24^ The SDMT involves pairing 9 abstract geometric symbols with single-digit numbers in a key, and orally stating the correct numbers for unpaired symbols as quickly as possible for 90 seconds. The primary outcome of the SDMT was the number of correct responses provided in 90 seconds (ie, raw score).

The CVLT-II involves an examiner reading aloud a list of 16 words (4 items belonging to 4 categories, such as vegetables, animals, furniture, and modes of transportation) that are randomly arranged; this was done 5 times in the same order at a rate of approximately 1 word per second. Participants recalled as many items as possible, in any order, following each reading of the list. The primary outcome of the CVLT-II was the total number of correct words identified over the 5 trials (ie, raw score).

The BVMT-R involved 3 trials of the examiner presenting a 2 × 3 array of abstract geometric figures approximately 16 inches in front of the participant for 10 seconds. The array was then removed, and participants drew the array as precisely as possible with the figures in the correct location. Each drawing was scored based on accurately portraying each figure and in its correct location using a 0–2 scale. The primary outcome of the BVMT-R was the total raw score across the 3 trials.

#### Demographic/clinical variables

We included demographic and clinical variables to describe the overall sample and 3 age-group subsamples. The demographic variables included age (y), sex, and race. The clinical variables included disease duration (y), MS type, and the Patient-Determined Disease Steps scale as a measure of mobility disability.^25^ The Patient-Determined Disease Steps is an 8-point, self-report scale with scores that range from 0 (no disability) to 8 (bedridden), and strongly correlates with the criterion standard Expanded Disability Status Scale.^25^

### Data analyses

The data analyses were performed using IBM SPSS Version 29.0.2.0 for the iOS platform.^a^ We provided descriptive values for continuous variables as mean (SD), ordinal variables as median (interquartile range), and categorical variables as number (percentage). We compared the 3 age groups for differences in MSWS-12 scores and performance-based walking outcomes using a linear trend analysis. Linear trend analysis is a direct approach within the analysis of variance family for directly testing the fit of a linear or other pattern of differences in scores on a measure among groups. This analysis tested the hypothesis of known-group or expected differences as a source of evidence for the construct validity of MSWS-12 scores (ie, we expected a linear increase or worsening in scores on the MSWS-12 across the young, middle-aged, and older adult groups that would parallel the worsening of scores on the T25FW, 6MW, and TUG among the age groups). We then examined bivariate correlations between MSWS-12 scores with performance-based walking outcomes (T25FW, 6MW, and TUG) and neurocognitive outcomes (BICAMS) using Pearson product-moment correlation coefficients. This analysis was done in the overall sample and the 3 separate age groups for providing convergent (ie, strong correlations with T25FW, 6MW, and TUG) and divergent (ie, small or moderate correlations with BICAMS) evidence for the construct validity of MSWS-12 scores. The final analysis involved partial correlations between MSWS-12 scores with performance-based walking outcomes (T25FW, 6MW, and TUG), controlling for neurocognitive outcomes (BICAMS) using partial Pearson product-moment correlation coefficients. This analysis was done in the overall sample and the 3 separate age groups to confirm that neurocognitive status was not a source of bias in the construct validation of MSWS-12 scores (ie, the correlations between MSWS-12 and T25FW, 6MW, and TUG were not dependent on BICAMS scores).

## Results

### Descriptive statistics

The descriptive statistics for the demographic, clinical, and neurocognitive variables for the overall sample and separately for the 3 age groups are provided in table 1.

### Known-group differences

The descriptive statistics for MSWS-12 scores and performance-based walking outcomes for the overall sample and the 3 separate age groups are provided in table 2. The linear trend analyses indicated similar worsening of walking outcomes across the 3 age groups for MSWS-12 (F_1,187_=12.7, *P*<.001, *ɳ*^2^=0.07), T25FW (F_1,187_=11.5, *P*<.001, *ɳ*^2^=0.06), 6MW (F_1,187_=13.1, *P*<.001, *ɳ*^2^=0.08), and TUG (F_1,184_=10.8, *P*<.001, *ɳ*^2^=0.06) scores. This provided known-group validity evidence for MSWS-12 scores, whereby MSWS-12 scores worsened with increasing age groups in a pattern consistent with worsening of performance on T25FW, 6MW, and TUG with increasing age groups.

### Bivariate correlations

The bivariate correlations between MSWS-12 scores with performance-based walking outcomes and neurocognitive outcomes for the overall sample and the 3 separate age groups are provided in table 3. As hypothesized, MSWS-12 scores had strong correlations with T25FW, 6MW, and TUG in the overall sample, and this was consistent across the 3 age groups. As hypothesized, MSWS-12 scores had weak or moderate associations with neurocognitive outcomes in the overall sample, and this was consistent across the 3 age groups. This pattern of correlations was consistent with our nomological network and provided convergent and divergent evidence of construct validity, whereby MSWS-12 more strongly correlated with walking (convergence) than neurocognitive (divergence) performance outcomes overall and across the 3 separate age groups.

### Partial correlations

The partial correlations between MSWS-12 scores with performance-based walking outcomes, controlling for neurocognitive outcomes, for the overall sample and the 3 separate age groups are provided in table 4. As hypothesized, MSWS-12 scores had strong partial correlations with T25FW, 6MW, and TUG in the overall sample and across the 3 age groups, even when controlling for neurocognitive outcomes. This provided further evidence of construct validity, whereby MSWS-12 scores were associated with performance-based walking outcomes independent of neurocognitive performance outcomes overall and across the 3 age groups.

## Discussion

The validation of inferences from scores on PROMs is an ongoing and evolving process,^19^ and this study adopted a historically derived framework involving a nomological network and hypothesis testing as fundamentals of establishing the construct validity from test scores^19–23^ when examining the construct validity of MSWS-12 scores among young, middle-aged, and older adults with MS. Overall, our results were consistent with hypotheses and indicated that (a) MSWS-12 scores differed across the samples of young, middle-aged, and older adults with MS in a similar manner as the performance-based walking outcomes (T25FW, 6MW, and TUG); (b) MSWS-12 scores were strongly associated with performance-based walking outcomes (T25FW, 6MW, TUG), but not neurocognitive outcomes (BICAMS), overall and consistently across the samples of young, middle-aged, and older adults with MS; and (c) neurocognitive performance did not influence the associations between MSWS-12 scores and performance-based walking outcomes (T25FW, 6MW, TUG) overall and across the 3 age-group samples. This strengthens the evidence for the construct validity of MSWS-12 scores as a measure of walking dysfunction across the adult lifespan among people with MS and particularly supports the application of this scale in clinical research and practice involving older adults with MS who are experiencing the combined effects of advanced age and MS disease progression. Our results support the value of the MSWS-12 as a screening instrument, descriptive measure, and outcome within clinical trials and practice that focus on monitoring walking from the patient’s perspective across the lifespan.

One approach for establishing the construct validity of scores on a PROM involves the comparison of mean scores between groups that differ in a known or established manner.^19^ This was done in the current study based on comparing MSWS-12 scores among the 3 age-group samples that concurrently differed in walking performance outcomes. As expected, we observed that the 3 age-group samples differed in MSWS-12 scores, and there were concurrent differences in T25FW, 6MW, and TUG performance. Indeed, scores on the MSWS-12 and performance-based walking outcomes worsened in a similar manner across the samples of young, middle-aged, and older adults with MS. Because the 3 age groups differed in walking performance, the observation that MSWS-12 scores differed in a concurrent manner would support known-group differences as an approach for construct validity of scores.

Another approach for establishing the construct validity of scores on a PROM involves the pattern of correlations with other measures, including those of similar (convergent) and dissimilar (divergent) constructs. This was done in the current study by examining the pattern of correlations between MSWS-12 scores with walking (T25FW, 6MW, TUG) and neurocognitive (SDMT, CVLT-II, BVMT-R from BICAMS) performance outcomes in the overall sample and the 3 separate age groups (young, middle-aged, and older adults). We observed strong associations between MSWS-12 scores with performance-based walking outcomes (T25FW, 6MW, TUG) in the overall sample and the 3 age groups (young, middle-aged, older adults), yet the correlations were weaker between the MSWS-12 scores and neurocognitive performance outcomes (SDMT, CVLT-II, BVMT-R). The observed pattern of correlations is consistent with our nomological network and existing construct validation research in younger samples of people with MS,^12,13,26,27^ but now extends that evidence across the adult lifespan, notably in older adults with MS.

There is considerable importance in controlling for variables that might influence or bias the construct validity of scores from a PROM. One such variable involves neurocognitive function because this can influence the accurate processing, recall, and reporting of past experiences, such as with walking. There is further evidence of cognitive–motor coupling in MS.^10,16,17^ To that end, we controlled for neurocognitive function when examining the construct validity of MSWS-12 scores by running partial correlations between MSWS-12 scores with performance-based walking outcomes (T25FW, 6MW, TUG) while controlling for scores from neurocognitive assessments (SDMT, CVLT-II, BVMT-R). We observed strong associations between MSWS-12 scores with performance-based walking outcomes (T25FW, 6MW, TUG) in the overall sample and the 3 age groups (young, middle-aged, older adults), even when controlling for neurocognitive outcomes (SDMT, CVLT-II, BVMT-R). This observed pattern of results is consistent with our nomological network and previous research involving younger samples with MS,^13^ but now extends the evidence that cognitive function does not bias the construct validity of the MSWS-12 scores across the adult lifespan, notably in older adults with MS.

### Study limitations

There are important limitations of our study. One limitation of the study is the cross-sectional, comparative design that does not permit examining the validity and meaning of changes in MSWS-12 scores over time among the 3 age groups, notably in older adults with MS over long periods of time (eg, decades). We did not include other correlates of walking and MSWS-12 scores, such as symptoms, physical activity, symptomatic medications, comorbidities, or MS characteristics, in our analysis. We further did not include social determinants of health (eg, race, ethnicity, employment, education) in our analyses. There is no control sample for comparison, yet this would be inappropriate given that the items on the MSWS-12 are specific to MS and seeking responses from a control sample without MS would present cognitive dissonance among a non-MS sample.

## Conclusions

Overall, we provide important new evidence for the construct validity of MSWS-12 scores as a measure of walking dysfunction across the adult lifespan in MS. Our results, in particular, support the application of the MSWS-12 as a PROM in clinical research and practice involving older adults with MS who experience the combined effects of advanced age and MS disease progression. Our results support the value of the MSWS-12 as a screening instrument, descriptive measure, and outcome within clinical trials and practice that focus on monitoring walking from the patient’s perspective across the lifespan and notably among older adults with MS.

## Supplier

a. SPSS, version 29.0; IBM.

## Figures and Tables

**Table 1:** Descriptive statistics for demographic, clinical, and neurocognitive variables for the overall sample and the 3 adult age groups.

Variable	Overall (N=187)	Young (n=50)	Middle-Aged (n=83)	Older (n=54)
Age (y)	50.1 (13.2)	33.2 (4.6)	49.5 (5.7)	65.7 (4.5)
Sex (n/% Female)	146/77.2%	38/77.6%	63/75.9%	45/78.9%
Race (n/% White)	125/66.1%	23/46.9%	54/65.1%	48/84.2%
MS type (n/% RRMS)	165/89.2%	44/93.6%	74/90.2%	47/83.9%
Disease duration (y)	13.0 (9.6)	5.7 (5.2)	11.7 (7.7)	21.3 (9.2)
PDDS (0–8)	1 (3)	1 (3)	1 (3)	3 (3)
SDMT (0–110)	49.8 (12.3)	54.4 (11.9)	51.7 (11.1)	42.9 (11.5)
CVLT-II (0–80)	45.3 (10.2)	46.8 (9.9)	46.8 (10.6)	42.2 (9.3)
BVMT-R (0–36)	21.8 (6.9)	24.9 (6.3)	22.5 (6.3)	18.1 (6.9)

Values represent mean (SD) for age, disease duration, and neurocognitive variables, and median (interquartile range) for PDDS. BVMT-R, Brief Visuospatial Memory Test-Revised; CVLT-II, California Verbal Learning Test-II; MS, multiple sclerosis; PDDS, Patient-Determined Disease Steps scale; RRMS, relapsing−remitting multiple sclerosis.

**Table 2:** Descriptive statistics for MSWS-12 scores and performance-based walking outcomes for the overall sample and the 3 adult age groups.

Variable	Overall (N=187)	Young (n=50)	Middle-Aged (n=83)	Older (n=54)
MSWS-12 (0–100)	30.8 (28.7)	22.5 (26.4)	29.0 (28.4)	41.2 (28.7)
T25FW (s)	5.4 (2.7)	4.7 (1.6)	5.3 (2.7)	6.3 (3.3)
6MW (ft)	1481 (446)	1591 (350)	1528 (465)	1307 (451)
TUG (s)	8.4 (4.5)	7.3 (3.3)	8.0 (3.4)	10.1 (6.3)

Values represent mean (SD). MSWS-12, 12-item Multiple Sclerosis Walking Scale; TUG, timed Up and Go test; T25FW, timed 25-foot walk; 6MW, 6-minute walk.

**Table 3:** Bivariate correlations between MSWS-12 scores, performance-based walking outcomes, and neurocognitive performance outcomes for the overall sample and the 3 adult age groups.

Variable	Overall (N=187)	Young (n=50)	Middle-Aged (n=83)	Older (n=54)
T25FW (s)	0.688^†^	0.684^†^	0.682^†^	0.691^†^
6MW (ft)	−0.751^†^	−0.728^†^	−0.730^†^	−0.755^†^
TUG (s)	0.665^†^	0.702^†^	0.649^†^	0.672^†^
SDMT (0–110)	−0.498^†^	−0.525^†^	−0.524^†^	−0.279*
CVLT-II (0–80)	−0.363^†^	−0.513^†^	−0.327^†^	−0.176
BVMT-R (0–36)	−0.265^†^	−0.084	−0.222*	−.0230

Values are presented as Pearson correlation coefficients. BVMT-R, Brief Visuospatial Memory Test-Revised; CVLT-II, California Verbal Learning Test-II; SDMT, Symbol Digit Modalities Test; TUG, timed Up and Go test; T25FW, timed 25-foot walk; 6MW, 6-minute walk.

* *P*<.05.

† *P*<.01.

**Table 4:** Partial correlations between MSWS-12 scores with performance-based walking outcomes, controlling for neurocognitive performance outcomes for the overall sample and the 3 adult age groups.

Variable	Overall (N=187)	Young (n=50)	Middle-Aged (n=83)	Older (n=54)
T25FW (s)	0.590*	0.533*	0.539*	0.669*
6MW (ft)	−0.664*	−0.599*	−0.644*	−0.737*
TUG (s)	0.550*	0.571*	0.490*	0.640*

Values are presented as partial Pearson correlation coefficients. Partial correlations controlling for SDMT, California Verbal Learning Test-II, and Brief Visuospatial Memory Test-Revised scores. TUG, timed Up and Go test; T25FW, timed 25-foot walk; 6MW, 6-minute walk.

* *P*<.01.

## List of abbreviations:

BICAMS: Brief International Cognitive Assessment for MS
BVMT-R: Brief Visuospatial Memory Test-Revised
CVLT-II: California Verbal Learning Test-II
MS: multiple sclerosis
MSWS-12: 12-item Multiple Sclerosis Walking Scale-12
PROM: patient-reported outcome measure
SDMT: Symbol Digit Modalities Test
TUG: timed Up and Go
T25FW: timed 25-foot walk
6MW: 6-minute walk
